# Curcumin inhibits lipolysis via suppression of ER stress in adipose tissue and prevents hepatic insulin resistance[S]

**DOI:** 10.1194/jlr.M067397

**Published:** 2016-07

**Authors:** Lulu Wang, Bangling Zhang, Fang Huang, Baolin Liu, Yuan Xie

**Affiliations:** Jiangsu Key Laboratory of Traditional Chinese Medicine Evaluation and Translational Research, Department of Pharmacology of Chinese Materia Medica,* China Pharmaceutical University, Nanjing, China; State Key Laboratory of Natural Medicines,† China Pharmaceutical University, Nanjing, China; Key Laboratory of Drug Metabolism and Pharmacokinetics,§ China Pharmaceutical University, Nanjing, China

**Keywords:** mitogen-activated protein kinase, lipolysis, adenosine 3′,5′-cyclic monophosphate, insulin resistance, gluconeogenesis, endoplasmic reticulum

## Abstract

Curcumin is natural polyphenol with beneficial effects on lipid and glucose metabolism and this study aimed to investigate the effects of curcumin on lipolysis and hepatic insulin resistance. Endoplasmic reticulum (ER) stress and lipolysis signaling in adipose and FFA influx, lipid deposits, and glucose production in liver were examined. Palmitate challenge and high-fat diet feeding evoked ER stress-associated lipolysis with cAMP accumulation in adipose tissue. Curcumin treatment inhibited adipose tissue ER stress by dephosphorylation of inositol-requiring enzyme 1α and eukaryotic initiation factor 2α and reduced cAMP accumulation by preserving phosphodiesterase 3B induction. Knockdown of mitogen-activated protein kinase α1/2α with siRNAs diminished such effects of curcumin. As a result from downregulation of cAMP, curcumin blocked protein kinase (PK)A/hormone-sensitive lipase lipolysis signaling, and thereby reduced glycerol and FFA release from adipose tissue. Curcumin reduced FFA influx into the liver by blocking FFA trafficking, and then prevented diacylglycerol deposits and PKCε translocation in the liver, resultantly improving insulin action in the suppression of hepatic gluconeogenesis. Curcumin decreased adipose lipolysis by attenuating ER stress through the cAMP/PKA pathway, reduced FFA influx into the liver by blocking FFA trafficking, and thereby improved insulin sensitivity to inhibit hepatic glucose production. These findings suggested a novel pathway of curcumin to prevent lipid deposits and insulin resistance in liver by beneficial regulation of adipose function.

Adipose tissue functions as a site for fat storage, whereas adipose dysfunction and increased ectopic fat deposition in the heart, liver, or muscle are considered to be responsible for cardiovascular risk factors and metabolic disorders, including obesity, diabetes, and insulin resistance. The action of insulin in liver is to suppress hepatic glucose production and hepatic insulin resistance, which is characterized by a decrease in the ability of insulin to suppress hepatic gluconeogenesis. Recently, Perry et al. (1) demonstrated that adipose lipolysis-induced fat deposition in liver induces hepatic insulin resistance, elucidating the functional interaction between adipose dysfunction and insulin resistance in the liver. Although the association of insulin resistance with inflammation and dysregulation of adipokine expression in adipose tissue has been well-documented (2, 3), accumulating evidence demonstrates the critical role of endoplasmic reticulum (ER) stress in adipose dysfunction (4–6). The ER functions in synthesizing, folding, and transporting proteins and also is the site of triglyceride synthesis and nascent lipid droplet formation. Misfolding proteins accumulate in the ER lumen, leading to the unfolded protein response through the activation of the canonical sensors, PERK, IRE1, and ATF6, in an attempt to restore ER homeostasis (7, 8). However, uncontrolled or sustained unfolded protein response evokes ER stress and induces metabolic disturbances in special tissue or cells, such as observed in liver, islet β cells, brain, and white adipose tissue (4, 9–11). Additionally, ER stress has been shown to induce inflammatory cascades and insulin resistance in adipocytes and trigger lipolysis in cultured adipocytes, and research has revealed that adipose tissue lipolysis in response to ER stress is mediated via cAMP/protein kinase (PK)A and ERK1/2 signaling (12–14). These events suggest that ER stress acts as a causal factor for the initiation of inflammation and lipolysis during adipose dysfunction and contributes to insulin resistance.

Intracellular cAMP acts as a second messenger for hormone-sensitive lipolysis and its formation and degradation are regulated by adenylate cyclase (AC) and cyclic nucleotide phosphodiesterases (PDEs), respectively. PDE3B is a member of the PDE superfamily and is mainly expressed in cells of importance for the regulation of lipid and glucose metabolism. Generally, PDE3B is affected by thyroid hormones, intracellular calcium concentration, and TNF-α and NF-κB activation (15, 16). In response to cAMP accumulation, PKA and subsequent hormone-sensitive lipase (HSL) activation initiate lipolysis in adipose and the increased FFAs released are responsible for elevated levels of circulating FFAs. Trafficking FFAs are taken up by the liver through special transport proteins, leading to an increase of hepatic triacylglycerol and diacylglycerol (DAG) concentrations. As an intermediate in the process of esterification, DAG accumulation induces PKCε translocation to the plasma membrane to bind and inhibit the activity of the insulin receptor, and then attenuates the action of insulin in suppression of gluconeogenesis by impairing insulin PI3K/Akt signaling, leading to increased hepatic glucose production (17–19). Despite the fact that the association of FFA flux with hepatic steatosis and impaired insulin sensitivity has been well-documented (20), the FFA/DAG pathway presents an alternative route for the development of hepatic insulin resistance and well demonstrates the contribution of adipose lipolysis to hepatic gluconeogenesis due to adipose dysfunction.

Curcumin is a natural polyphenolic compound presented in *Curcuma longa*. Curcumin exerts a wide range of bioactivities implicated in anti-tumor inflammation inhibition and neuroprotection (21–23), and its beneficial effects on the management of metabolic disorders has recently received great attention. Curcumin has been documented to lower blood glucose by reducing hepatic glucose production and sensitizing insulin action through different pathways in experimental models (24, 25). In addition, curcumin attenuates lipolysis in adipocytes and shows positive influence on weight management in overweight people (26, 27). In view of the beneficial effects of curcumin on the regulation of glucose and lipid homeostasis, we raised the hypothesis that blocking FFA trafficking from adipose tissue to the liver might be a working pathway for curcumin in the prevention of hepatic insulin resistance. Our work in high-fat diet (HFD)-fed mice is aimed to prove that curcumin inhibits lipolysis in adipose tissue by suppression of ER stress via regulation of mitogen-activated PK (AMPK) activity, and thus improves insulin signaling and reduces glucose production in the liver by blocking DAG-induced PKCε translocation.

## MATERIALS AND METHODS

### Materials

Curcumin (purity ≥98%) was purchased from Nanjing Zelang Medical Technology Co., Ltd. (Nanjing, China). Compound C, thapsigargin (Thaps), and tauroursodeoxycholic acid (TUDCA) were obtained from Sigma (St. Louis, MO). Tunicamycin (ab120296) and forskolin (ab120058) were from Abcam (Cambridge, MA). AICA riboside and PKA inhibitor H89 (S1643) were purchased from Beyotime (Beyotime Institute of Biotechnology, Shanghai, China). Palmitate (PA) was purchased from Sinopharm Chemical Reagent Co., Ltd. (Shanghai, China) and dissolved in ethanol to prepare 200 mM stock solution and then was further diluted with medium containing 10% FFA-free BSA (1:19) to obtain a concentration of 10 mM before use. The antibodies against phospho-HSL (Ser660), HSL, phospho-Akt (Thr308), AMPK (2532s), phospho-AMPK (T172) (2531s), and phospho-(Ser/Thr) PKA substrate were from Cell Signaling Technology (Beverly, MA) and antibodies for PCK1, CD36, phospho-inositol-requiring enzyme 1α (IRE1α) (S724), and IRE1α were from Abcam. The antibodies of G6Pase-α (H-60) AMPKα1/2, and control siRNA and transfection reagents were obtained from Santa Cruz Biotechnology, Inc. (Santa Cruz, CA). The antibodies of phospho-eukaryotic initiation factor 2α (eIF2α), eIF2α, PDE3B, goat anti-rabbit IgG (H+L) HRP, goat anti-rabbit IgG (H+L) HRP, GAPDH, Akt (A444), and Na^+^/K^+^-ATPase α1 (G19) were from Bioworld Technology (St. Paul, MN).

### Animals and treatment

Male C57BL/6 mice (6–8 weeks old) were acclimatized with 12 h dark-light cycles under a constant temperature (22 ± 2°C) and had free access to water and food. All the experiments were carried out in accordance with the internationally accepted guide for the care and use of laboratory animals and were approved by the Animal Ethics Committee of the School of Chinese Materia Medica, China Pharmaceutical University.

Mice were fed with HFD (10% lard, 10% yolk, 1% cholesterol, 0.2% cholate, and 78.8% standard diet; Nanjing Qinglongshan Experiment Animal Center) for 10 days to build an early stage of insulin resistance model. In the meantime, curcumin (50 mg kg^−1^) and TUDCA (50 mg kg^−1^) were given intragastrically. At the end of HFD feeding, mice were fasted for 8–10 h before collecting blood from the orbital sinus. Levels of glucose, triglyceride, total cholesterol, and FFAs in the blood were assayed with commercial kits. Mice were fasted for 8 h and euthanized by cervical dislocation. Epididymis adipose tissue and livers were isolated and stored at −80°C for assay

### Treatment in adipose tissue

Normal mice were euthanized by cervical dislocation and epididymis adipose tissue was isolated and rinsed with cold PBS. Adipose tissue was cut into pieces and incubated with PA (100 μM) with the presence of curcumin (0.1, 1, 10 μM) or TUDCA (100 μM) for 24 h. The adipose tissue was homogenized in PBS, and AMP and cAMP contents in the suspension were measured by ELISA kits (Chengbin Biotech, Shanghai, China). To exclude the potential influence of PA presented in the medium on the determination of FFAs, adipose tissue was pretreated with curcumin and TUDCA at given concentrations and then stimulated with PA (100 μM) for 2 h. After washing, the adipose tissue was incubated for another 22 h. FFA and glycerol contents in the medium were measured with commercial kits.

### Preparation of adipose tissue-conditioned medium from HFD-fed mice

Epididymis adipose tissue was isolated from HFD-fed mice and cut into small pieces, then incubated in DMEM for 24 h; adipose tissue of normal mice was treated with PA (100 μM) for 2 h, and after washing, the adipose tissue was incubated in DMEM for another 22 h. The supernatant was harvested as conditioned medium (CM) derived from adipose tissue [adipose tissue-CM (AT-CM)], and then kept at −70°C for further study. Meanwhile, we prepared CM from 3T3-L1 cells when AMPKα1/α1 was silenced by siRNA. For the preparation of forskolin-derived condition medium, the adipose tissue was incubated with forskolin (10 μM) for 24 h.

### Oral glucose load test

HFD-fed mice were fasted for 12 h and glucose (2 g/kg, po) was administered. Blood was collected from the orbital sinus at regular intervals after glucose load. Serum glucose was determined by a commercial kit based on the glucose oxidase peroxidase method. Blood glucose area under carve was calculated for blood glucose as follows: 0.25 × [Bg0 + Bg0.25]/2 + 0.25 × [Bg0.25 + Bg0.5]/2 + 0.5 × [Bg0.5 + Bg1.0]/2 + [Bg1 + Bg2]/2 (Bg0, Bg0.25, Bg0.5, Bg1.0, and Bg2.0 refer to the blood glucose concentration at 0, 0.25, 0.5, 1.0, and 2.0 h after the glucose load).

### Hepatic lipid accumulation and glucose production

HFD-fed mice were euthanized by cervical dislocation and the livers were removed and homogenized in PBS. DAG contents in the supernatant were measured using a commercial kit (Dizhao, Nanjing. China). Triglyceride was assayed using a triglyceride assay kit (GPO-POD; Applygen Technologies Inc., Beijing, China). For hepatic glucose production, primary hepatocytes were freshly isolated from mice (fasting over 18 h) by collagenase digestion of the liver and cultured overnight in recovery medium (DMEM) supplemented with 15% (v/v) fetal bovine serum, 100 μg/ml streptomycin, and 100 U/ml penicillin at 37°C in a 5% CO_2_ atmosphere. After washing, the medium was replaced by 200 μl of glucose production medium consisting of KRH [containing 118 mM NaCl, 5 mM KCl, 1.3 mM CaCl_2_, 1.2 mM MgSO_4_, 1.2 mM KH_2_PO_4_, and 30 mM HEPES containing 0.5% BSA (pH 7.4)] supplemented with 10 mM sodium pyruvate (Generay Biotech) and 100 nM insulin. Glucose contents in the medium after 4 h were tested by a commercial kit.

### Evaluation of PDE activity in adipose tissue

Epididymis adipose tissue was isolated from HFD-fed mice and homogenized in double-distilled water. The supernatant was desalted by gel filtration and then measured using a PDE activity assay kit (Colorimetric) (Abcam; ab139460).

### Western blot

The protein from tissue or cell samples was electrophoresed on SDS-PAGE, transferred to PVDF membranes (Millipore Co., Ltd.), and blocked at room temperature for 2 h. The blots were incubated with primary antibodies at 4°C overnight, washed with PBS three times, and then incubated with the secondary antibody at room temperature for 2 h or at 4°C overnight. The immunoblots were detected by ECL Western blot detection system. To determine the membrane PKCε, membrane protein was prepared with a membrane protein extraction kit (Sangon Biotech, Shanghai, China) according to the manufacturer’s instructions.

### AMPKα siRNA transfection

The 3T3-L1 cells (Chinese Academy of Sciences, Shanghai, China) were cultured with siRNA duplex specific AMPKα1/2 (sc-45312; Santa Cruz Biotechnology) or control siRNA (sc-37007; Santa Cruz Biotechnology) using transfection reagents (sc-29528; Santa Cruz Biotechnology) following the manufacturer’s instructions. After 5–7 h of transfection, the medium was replaced, and 48 h later cells were treated with or without PA (100 μM) or Thaps (1 μM) for 24 h. The specificity of interference was confirmed by Western blot.

### Statistics

At least three separate experiments were performed and all the results were expressed as mean ± SD. Data were analyzed by using a two-tailed *t*-test or a one-way ANOVA test with a Student-Newman-Keuls test for comparison of two groups. Values of *P* < 0.05 were considered statistically significant.

## RESULTS

### Curcumin suppressed ER stress in mouse adipose tissue

**Fig. 1.**
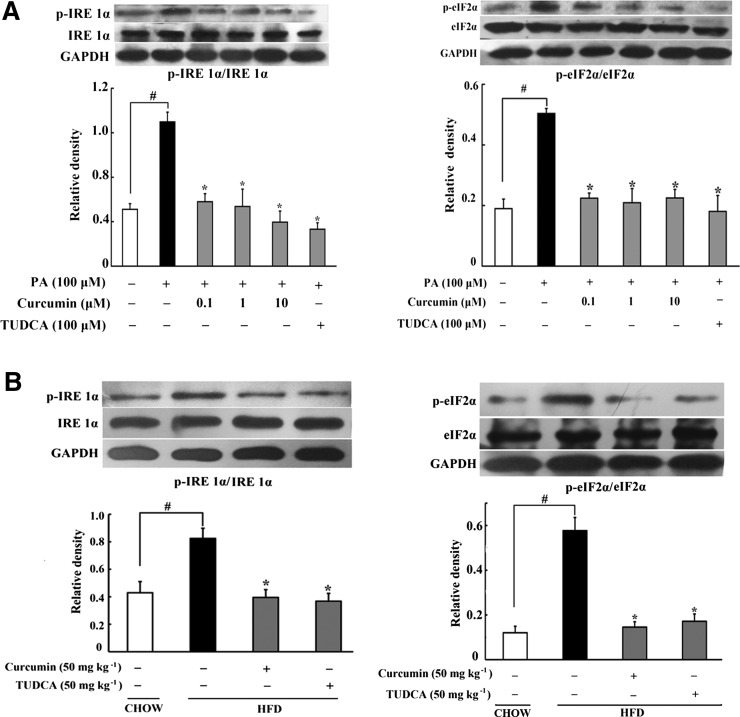
Curcumin suppressed ER stress in adipose tissue. A: Expressions of p-IRE1α/IRE1α and p-eIF2α/eIF2α in adipose tissue exposed to PA were detected by Western blot. B: p-IRE1α/IRE1α and p-eIF2α/eIF2α expression in adipose tissue of HFD-fed mice. The results are expressed as the mean ± SD of four separate experiments. *P < 0.05 versus model; ^#^*P* < 0.05 versus control.

Lipid is a major component of ER membrane, and its disturbance always leads to ER stress (28). Consistently, 100 μM PA induced apparent ER stress in mouse adipose tissue, evidenced by enhanced phosphorylation of IRE1α (p-IRE1α) and phosphorylation of eIF2α (p-eIF2α), while total IRE1α and eIF2α expression remained unchanged. Curcumin (0.1, 1, 10 μM) treatment inhibited IRE1α and eIF2α activation by dephosphorylation, indicative of the role in suppression of ER stress (**Fig. 1A**). As a positive control, the ER stress inhibitor, TUDCA, also effectively attenuated IRE1α and eIF2α phosphorylation.

Similarly, short-term HFD feeding (10 days) increased p-IRE1α and p-eIF2α expression in mouse adipose tissue, indicating ER stress in the early course of lipid disorders. Oral administration of curcumin (50 mg kg^−1^) and TUDCA (50 mg kg^−1^) reduced IRE1α and eIF2α phosphorylation, well demonstrating their inhibitory effects on ER stress in vivo (Fig. 1B).

### Curcumin blocked cAMP/PKA activation in adipose tissue

**Fig. 2.**
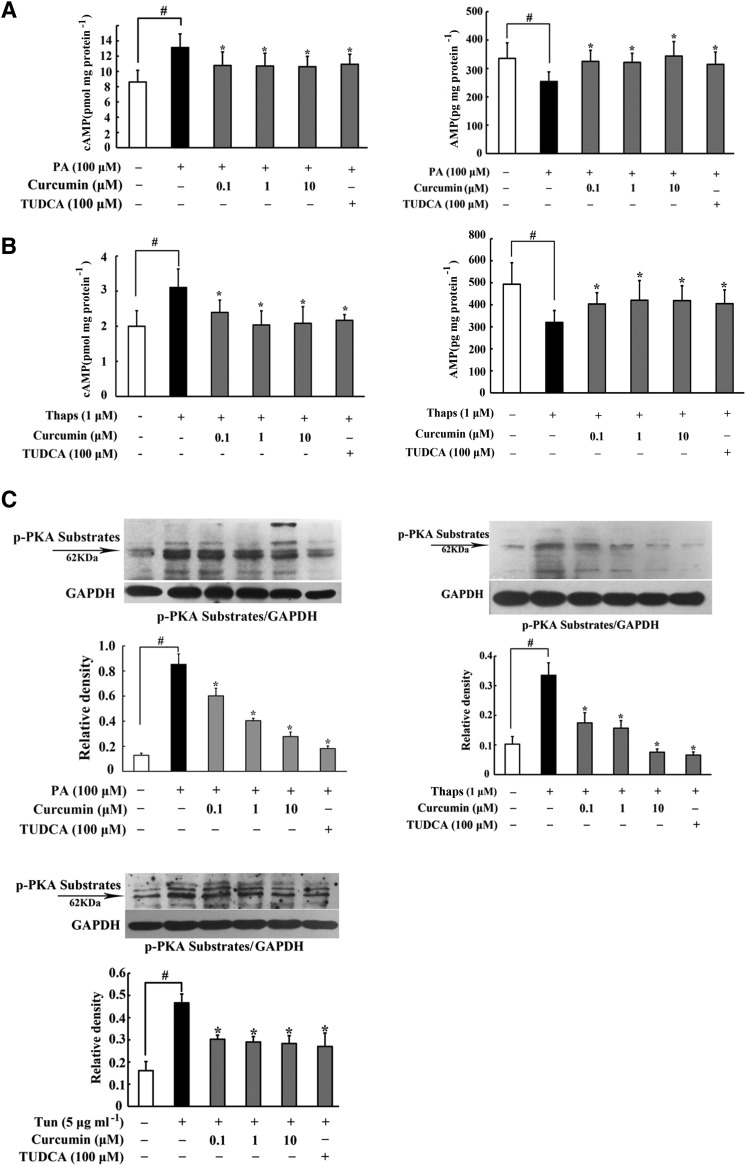

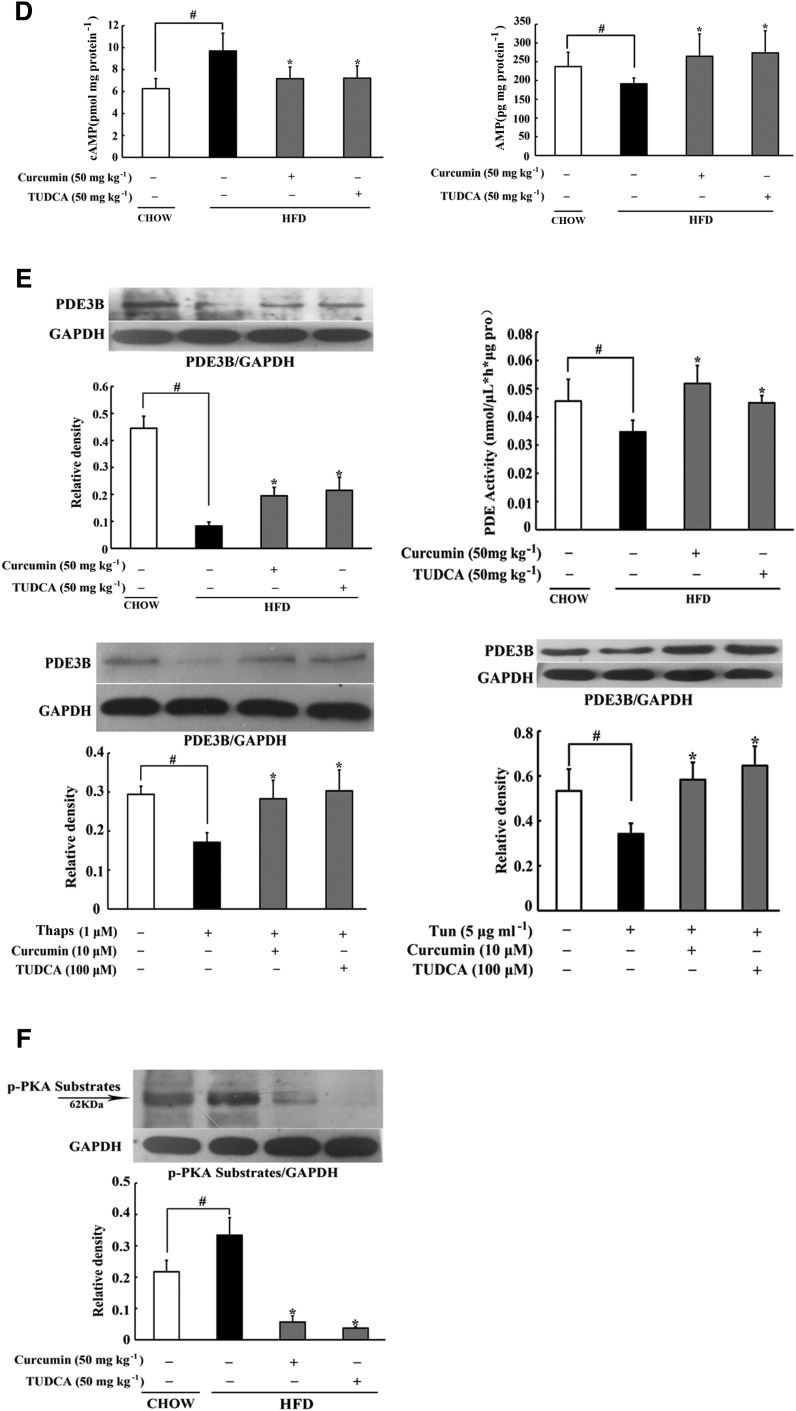
Curcumin blocked cAMP/PKA activation by restoring PDE3B. A, B: cAMP and AMP contents in adipose tissue incubated with PA or thapsigargin (Thaps) for 24 h were detected using ELISA (n = 6). C: Phosphorylation of PKA substrates in adipose tissue exposed to PA or Thaps and tunicamycin (Tun) for 24 h were examined by Western blot. D: The contents of cAMP and AMP in adipose tissue of HFD-fed mice were assayed used ELISA kits (n = 6). E: PDE3B protein expression in adipose tissue of HFD-fed mice or in adipose tissue from normal mice challenged with thapsigargin (1 μM) and tunicamycin (5 μg ml**^−1^**) for 24 h were examined by Western blot; PDE activity was measured using a PDE activity assay kit (n = 6). F: Phosphorylation of PKA substrates in adipose tissue of HFD-fed mice were examined by Western blot. The results are expressed as the mean ± SD of four independent experiments. *P < 0.05 versus model; ^#^*P* < 0.05 versus control.

PKA activation is an action response to the second messenger, cAMP. PA challenge increased cAMP production significantly, while the AMP content was reduced in adipose tissue; whereas these alterations were reversed by treatment with curcumin and TUDCA (**Fig. 2A**). Meanwhile, curcumin also effectively reduced cAMP accumulation and restored AMP contents in adipose tissue subjected to the ER stress inducer, Thaps (Fig. 2B). As a downstream result from prevention of cAMP accumulation, curcumin inhibited PA- and Thaps-induced PKA 62 KDa substrate phosphorylation in adipose tissue (Fig. 2C). Tunicamycin, another ER stress inducer, also evoked PKA 62 KDa substrate phosphorylation; whereas this action was abrogated by curcumin and TUDCA treatment (Fig. 2C). These results suggested that the inhibitory effect of curcumin on PKA activation was relative to suppression of ER stress.

Furthermore, we validated the effects of curcumin on the cAMP/PKA pathway in HFD-fed mice. Paralleled to in vitro results, we proved that curcumin lowered cAMP accumulation in adipose tissue in accordance with upregulation of AMP content (Fig. 2D). To explain the cause for the decrease of cAMP, we examined the expression of PDE3B, which hydrolyzed cAMP, and found that curcumin administration preserved PDE3B induction in adipose tissue of HFD-fed mice. In addition, a PDE3B enzymatic activity assay showed that HFD feeding attenuated PDE activity, while curcumin and TUDCA administration preserved PDE3B activity in adipose tissue of HFD-fed mice. Meanwhile, curcumin treatment also normalized PDE3B expression in adipose tissue exposed to Thaps and tunicamycin challenge (Fig. 2E). These results suggested that curcumin prevented cAMP accumulation by preserving PDE3B activity in adipose tissue. As an expected result, oral administration of curcumin prevented PKA activation in adipose tissue of HFD-fed mice (Fig. 2F).

### Curcumin inhibited adipose lipolysis

**Fig. 3.**
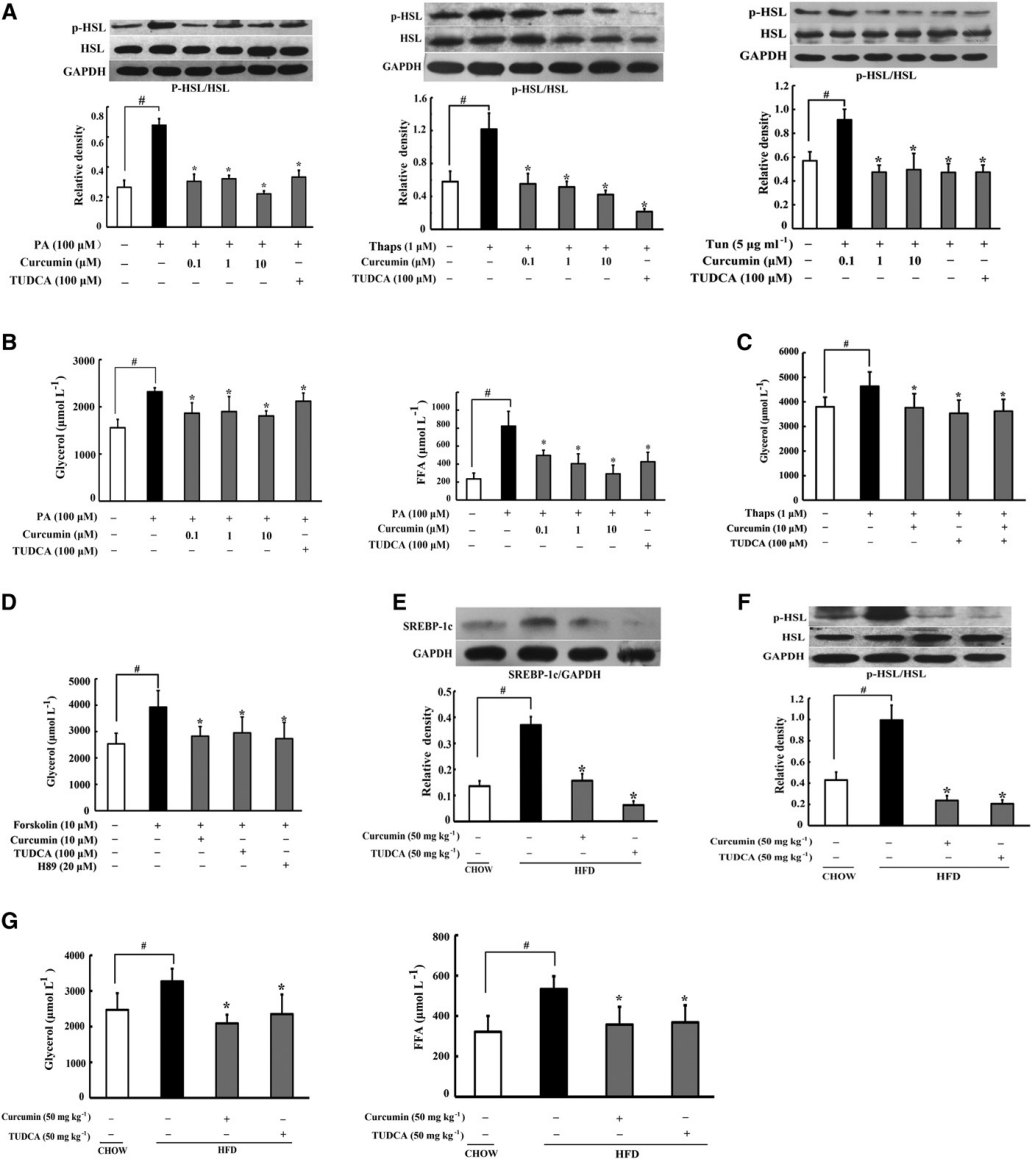
Curcumin inhibited adipose lipolysis. A: HSL phosphorylation in adipose tissue subjected to PA or thapsigargin (Thaps) challenge (24 h) were determined by Western blot. B: Glycerol and FFA release in adipose tissue treated with PA. C, D: Glycerol release in adipose tissue exposed to Thaps and forskolin for 24 h (n = 6). SREBP-1c (E) and p-HSL/HSL (F) in adipose tissue of HFD-fed mice were determined by Western blot. G: Glycerol and FFA contents in the AT-CM of HFD-fed mice (n = 6). Data are expressed as the mean ± SD of four independent experiments. *P < 0.05 versus model; ^#^*P* < 0.05 versus control.

HSL, in response to PKA, is the enzyme that hydrolyzes intracellular triacylglycerol and DAG, further influencing the release of glycerol and FFAs from adipocytes. PA, as well as ER inducer Thaps and tunicamycin, increased HSL phosphorylation in adipose tissue, but this action was blocked by curcumin and TUDCA (**Fig. 3A**). Therefore, curcumin and TUDCA inhibited PA-induced glycerol and FFA release from adipose tissue (Fig. 3B). Similarly, curcumin and TUDCA also reduced Thaps-induced glycerol release (Fig. 3C). Forskolin-stimulated glycerol release was also suppressed by curcumin and TUDCA treatment (Fig. 3D). Forskolin is an activator of AC, and PKA inhibitor H89 abolished its enhancing effect on glycerol release (Fig. 3D), indicating that curcumin inhibited lipolysis by blocking cAMP/PKA signaling.

HFD feeding caused SREBP-1c induction and a high level of HSL phosphorylation, suggesting that increased esterification and lipolysis occurs simultaneously in lipid disorders. Curcumin administration diminished SREBP-1c induction (Fig. 3E) and inhibited HSL activation by dephosphorylating (Fig. 3F), and thereby reduced glycerol and FFAs released from adipose tissue of HFD-fed mice (Fig. 3G), demonstrating its anti-lipolytic action in HFD mice. Similar to curcumin, oral administration of TUDCA also suppressed HSL activation and inhibited lipolysis in HFD-fed mice, indicating that ER stress was involved in lipolysis induced by HFD feeding.

### Curcumin reduced lipid deposits in the liver

**Fig. 4.**
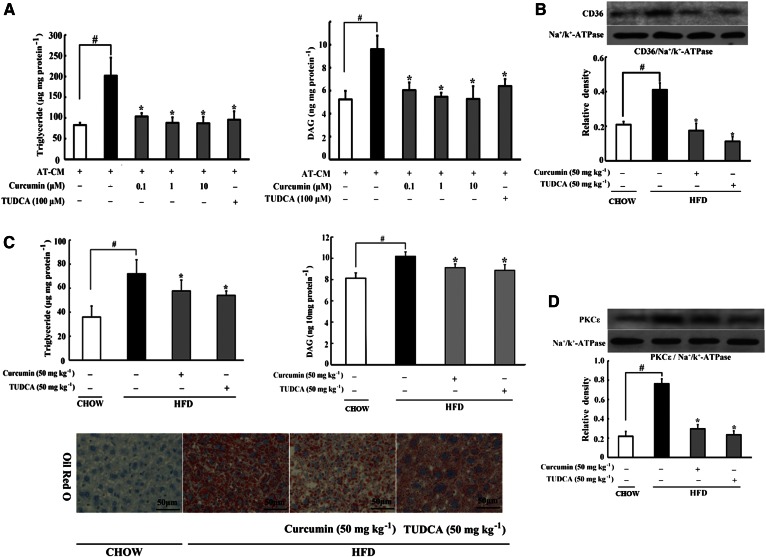
Curcumin reduced lipid deposits in the liver. A: Triglyceride and DAG accumulation in hepatocytes incubated with AT-CM for 24 h (AT-CM was prepared from PA-stimulated adipose tissue), n = 6. B: CD36 membrane protein expression in the liver of HFD-fed mice was determined by Western blot (n = 4). The contents of triglyceride (C) and DAG in liver of HFD-fed mice (n = 8) were determined using commercial kits. Liver Oil Red O staining was also detected. D: PKCε translocation in the liver was determined by Western blot (n = 4). Data are expressed as the mean ± SD (n = 8). *P < 0.05 versus model; ^#^*P* < 0.05 versus control.

To investigate ectopic lipid deposition in the liver, we prepared AT-CM from PA-stimulated adipose tissue to incubate with hepatocytes, and found that hepatocytes’ intracellular triglyceride and DAG levels elevated about two times, indicating lipid deposits in hepatocytes. Curcumin treatment with adipose tissue reduced triglyceride and DAG accumulation in hepatocytes (**Fig. 4A**), indicating that inhibition of lipolysis in adipose tissue prevented lipid deposits in the liver. Consistent with the elevated level of circulating FFAs, HFD feeding in mice led to increased CD36 expression and accumulation of triglyceride and DAG in the liver. Oral administration of curcumin reduced CD36 expression (Fig. 4B) and prevented triglyceride and DAG accumulation. Oil Red O staining examination also demonstrated that oral administration of curcumin and TUDCA effectively reduced lipid deposition in the liver (Fig. 4C). Curcumin reduced DAG accumulation and thereby prevented PKCε translocation to the cell membrane in the liver (Fig. 4D), indicating that curcumin prevented DAG-induced PKCε activation. TUDCA administration also effectively inhibited CD36 expression, reduced triglyceride and DAG contents, and suppressed PKCε activation in the liver of HFD-fed mice.

### Curcumin ameliorated insulin resistance in the liver

**Fig. 5.**
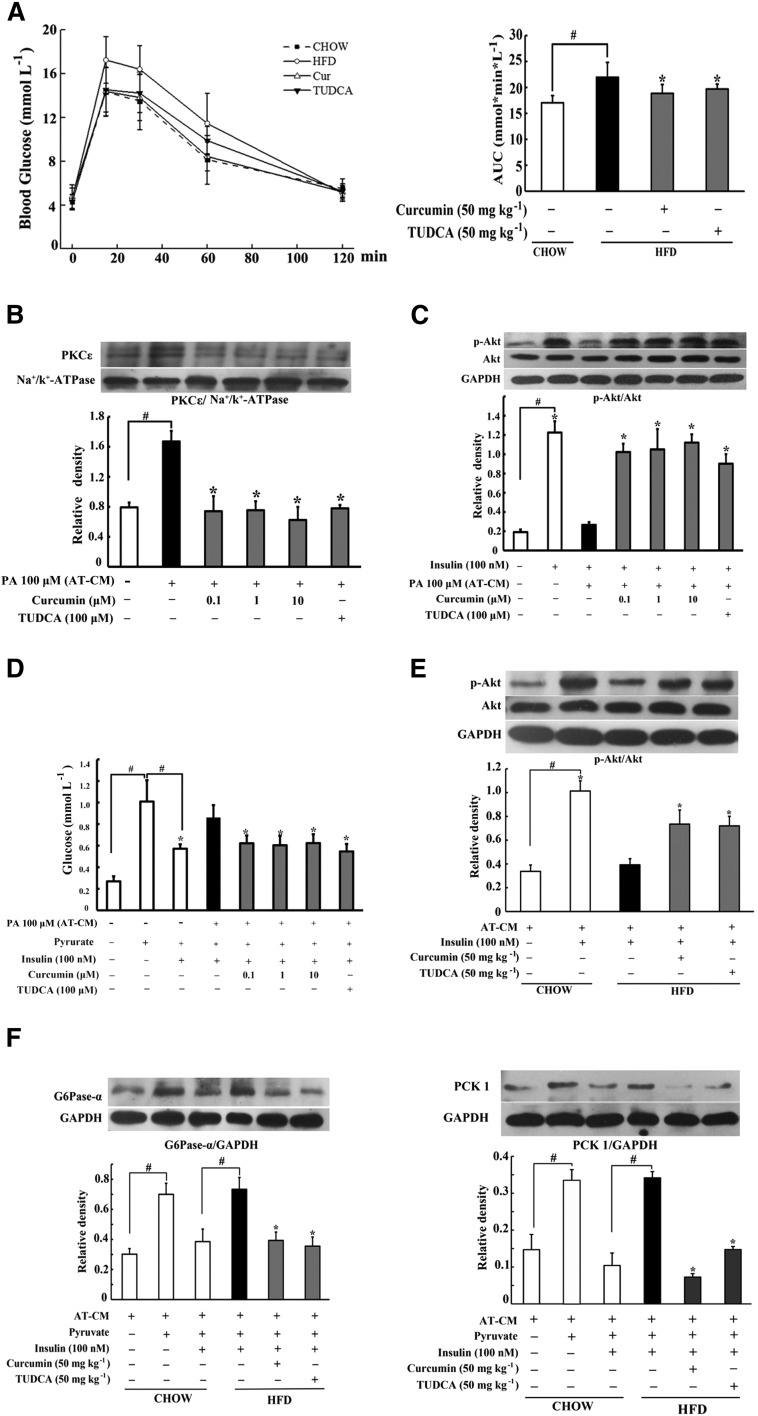
Curcumin improved insulin resistance in the liver of HFD-fed mice. Blood of HFD-fed mice was collected at regular intervals for glucose after the administration of 2 g kg^−1^ glucose. A: Glucose concentrations within 2 h after glucose load and AUC were calculated. The results are expressed as the mean ± SD (n = 10). Hepatocytes were incubated with PA-stimulated AT-CM for 24 h. PKCε translocation (B) and insulin-mediated Akt phosphorylation (C) in hepatocytes were detected by Western blot. D: Hepatic glucose production in the presence of pyruvate and insulin (n = 6). Hepatocytes were incubated with AT-CM derived from chow- or HFD-fed mice for 24 h. Akt phosphorylation (E) and induction for G6Pase-α and PCK1 (F) in the presence of insulin were detected by Western blot. The results are expressed as the mean ± SD of three independent experiments. *P < 0.05 versus model; ^#^*P* < 0.05 versus control.

Adipose dysfunction is associated with insulin resistance. Consistent with this, HFD feeding induced glucose intolerance in mice, indicated by delayed glucose disposal after oral glucose load. Oral administration of curcumin improved glucose tolerance by promoting glucose disposal (**Fig. 5A**), and reduced the elevated levels of blood FFAs and total cholesterol without affecting the level of triglyceride (supplementary Fig. 1A–C). Meanwhile, we also observed food intake and body weight gains in HFD mice, and no significant influence was found in curcumin treatment (supplementary Fig. 1D, E).

Incubation of hepatocytes with PA-stimulated AT-CM promoted PKCε translocation to the cell membrane and impaired insulin-mediated Akt phosphorylation (Fig. 5B, C), indicating the impaired insulin signaling. Curcumin treatment in AT-CM blocked PKCε translocation and thereby enhanced Akt phosphorylation in response to insulin (Fig. 5B, C). Moreover, curcumin treatment restored insulin action in the suppression of hepatic glucose production in response to pyruvate, a main substrate for gluconeogenesis (Fig. 5D). We prepared CM from the adipose tissue of HFD-fed mice to incubate hepatocytes, and found that curcumin administration during HFD feeding improved insulin-mediated Akt phosphorylation and effectively enhanced the inhibitory effects of insulin on G6Pase-α and PCK1 induction in hepatocytes (Fig. 5E, F). Together, these results indicated that curcumin ameliorated hepatic insulin resistance via reducing FFA flux into the hepatocytes.

### Curcumin inhibited ER stress and lipolysis via regulation of AMPK

**Fig. 6.**
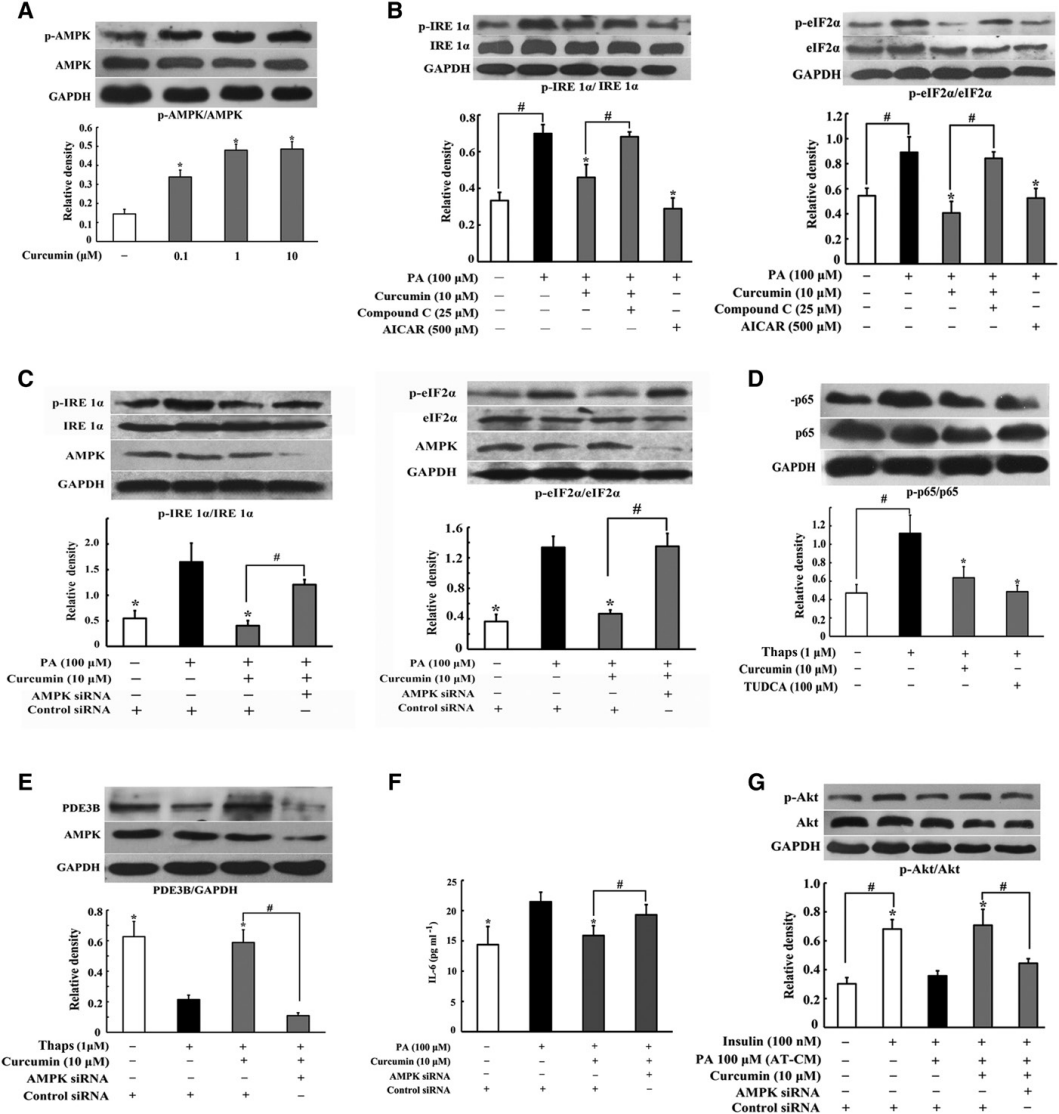
AMPK played an important role in the inhibition of ER stress and lipolysis by curcumin. Adipose tissues were incubated with curcumin for 4 h. A: AMPK phosphorylation was determined by Western blot. B: Adipose tissue was incubated with curcumin followed by stimulation with PA for 24 h; IRE1α and eIF2α phosphorylation was determined by Western blot. C: The 3T3-L1 cells were transfected with AMPKα1/α2 or control siRNAs; expression of p-IRE1α and p-eIF2α was determined by Western blot. D: The p65 phosphorylation in adipose tissue exposed to thapsigargin (Thaps) stimulation for 24 h was detected by Western blot. E: PDE3B expression in adipocytes subjected to Thaps challenge when AMPKα1/α2 or control siRNAs were silenced with siRNAs. F: The 3T3-L1 cells were transfected with AMPKα1/α2 or control siRNAs, content of IL-6 in the medium was detected by ELISA kit (n = 6) and CM was used to incubate hepatocytes for 24 h. G: Insulin-mediated Akt phosphorylation in hepatocytes was detected by Western blot. The results are expressed as the mean ± SD of three independent experiments. *P < 0.05 versus model; ^#^*P* < 0.05 versus indicated treatment.

AMPK is an energy sensor regulating glucose and lipid metabolism. Curcumin increased AMPK phosphorylation in adipocytes, demonstrating its action in promoting AMPK activity (**Fig. 6A**). The AMPK activator, AICA riboside, inhibited IRE1α and eIF2α phosphorylation and cotreatment with AMPK inhibitor compound C attenuated the inhibitory effects of curcumin on IRE1α and eIF2α (Fig. 6B), suggesting the involvement of AMPK. Then, we investigated the effect of curcumin on ER stress by knockdown of AMPKα1/α2 with siRNA and found that silencing of AMPK diminished the inhibitory effects of curcumin on IRE1α and eIF2α activation (Fig. 6C), indicative of the essential role of AMPK in curcumin action. ER stress evoked NF-κB activation, which suppresses PDE3B gene transcription (16, 29). Curcumin attenuated ER inducer Thaps-induced p-65 expression (Fig. 6D) and silencing of AMPK blocked the action of curcumin in preserving PDE3B induction (Fig. 6E). These results suggested that curcumin suppressed ER stress and preserved PDE3B by inhibition of NF-κB signaling in a manner dependent of AMPK.

Finally, we prepared PA-stimulated CM in AMPKα1/α2-knockdown 3T3-L1 cells, and used the CM to incubate hepatocytes to detect Akt expression. We found that the inhibitory effect of curcumin on IL-6 production was attenuated by knockdown of AMPK (Fig. 6F). Consistently, knockdown AMPK blocked the action of curcumin in normalizing p-Akt (Fig. 6G). These results suggested that AMPK activation in adipocytes contributed to curcumin for the amelioration of insulin resistance in the liver.

## DISCUSSION

In the present study, we found that short-term HFD feeding in mice induced adipose dysfunction and hepatic insulin resistance, indicating that insulin resistance occurs in the early stage of lipid disorders. In this model, lipid was deposited in the liver with elevated levels of circulating FFAs, while other metabolic parameters were unchanged. As enhanced lipolysis is responsible for the elevated levels of circulating FFAs, this model indicated the special role of FFAs in the development of insulin resistance and allowed us to investigate the direct impact of trafficking FFAs on hepatic glucose production in the setting of adipose dysfunction.

As lipid is a major component of ER membrane, its disturbance can pose challenges to induce ER stress (28). In the present study, PA challenge, as well as HFD feeding, evoked ER stress in adipose tissue, indicated by enhanced IRE1α and eIF2α phosphorylation. In addition to oxidative stress and inflammation, ER stress is implicated in lipolysis in adipocytes (13, 30). Although it is shown that ER stress initiates lipolysis through cAMP/PKA signaling, we showed that this action was relative to the regulation of PDE3B activity, as we observed cAMP accumulation with attenuated PDE3B expression and inhibited PDE activity in the adipose tissue of HFD-fed mice. PDE3B prevents cAMP accumulation by degradation, but its activation can be inhibited by inflammation, resulting in cAMP accumulation (15, 16). Given the association of inflammation with ER stress, the inhibitory effect of curcumin on ER stress should contribute to suppressing inflammation and restoring the ability of PDE3B. Indeed, we observed that curcumin treatment inhibited NF-κB activation and preserved PDE3B expression. In addition to being regulated by PDE3B, AMP exerts the ability to reduce cAMP production by inhibition of AC, and this regulation is relative with the reduced cellular energy charge (31, 32). Forskolin induces cAMP/PKA activation without affecting G-protein. Curcumin reduced forskolin-induced lipolysis, suggesting that its action is independent of G-protein regulation. Oral administration of curcumin increased AMP contents in adipose tissue of HFD-fed mice, and this action should work together with preserved PDE3B activation to prevent cAMP accumulation. As the results from upstream regulation, curcumin blocked PKA/HSL activation, and then effectively reduced glycerol and FFA release from adipose tissue. As an energy sensor, AMPK regulates energy metabolism responsible for cellular homeostasis and is documented to inhibit ER stress (33). Curcumin enhanced AMPK phosphorylation, and it was tempting to know whether this action contributed to the inhibition of lipolysis under ER stress conditions. “Silencing” of AMPK with siRNAs diminished its action in the regulation of ER stress and PDE3B, indicating that curcumin reduced cAMP accumulation by suppression of ER stress in a manner dependent on AMPK activation.

In diabetes, the elevated levels of fasting blood glucose are mainly due to augmented hepatic gluconeogenesis. Some studies demonstrate the contribution of FFA trafficking to hepatic glucose production and insulin resistance (17, 34). In the current study, short-term HFD feeding enhanced lipolysis from adipose tissue with impaired glucose tolerance. The level of circulating FFAs was elevated, while other metabolic parameters remained unchanged, suggesting the potential role of FFAs in insulin resistance. Oral administration of curcumin in HFD-fed mice inhibited lipolysis in adipose tissue and reduced lipid deposit in the liver with downregulation of CD36 expression in the membrane, indicating that the inhibitory effects of curcumin on adipose lipolysis prevented FFA flux into the liver. To confirm the influence of curcumin on the functional interaction between adipose dysfunction and hepatic insulin resistance, we prepared CM from adipose tissue to treat hepatocytes and well mimicked these alterations. DAG is an intermediate in the esterification process after FFA flux and is shown to activate PKC (35). Samuel et al. (36) evaluated PKC isoform activation with hepatic fat accumulation in rats and found increased PKCε translocation to the cell membrane. Inhibition of PKCε prevents hepatic insulin resistance in nonalcoholic fatty liver disease (17), further confirming the association of DAG deposit with insulin resistance. Curcumin inhibited DAG-associated PKCε activation, improved insulin signaling in hepatocytes, and thereby preserved insulin action in the suppression of gluconeogenesis by inhibiting G6P-α and PCK1 induction. These results well explained the beneficial effects of curcumin on the improvement of glucose tolerance in HFD-fed mice. Consistent with the situation in the liver, the association of DAG deposits in the muscle with insulin resistance is also observed in obese and diabetic individuals (37). In the past decades, people paid more attention to the association of oxidative stress and inflammation with insulin resistance in lipid disorders; the FFA/DAG/PKC pathway provides novel insight into the impact of lipid challenge on insulin resistance. Our data also raised the possibility that blocking the FFA trafficking from adipose tissue to the liver might be a potential therapeutic strategy for the prevention of insulin resistance.

It has been well-established that inflammation is tightly associated with insulin resistance, and this action is proposed to be mediated through the impairment of insulin receptor substrate-1 by inflammatory molecules (38). In the present study, ER stress-associated inflammation in adipose tissue induced insulin resistance in the liver via lipolysis, and this finding presented another way for inflammation-associated insulin resistance. AMPK enhances insulin sensitivity and regulates glucose homeostasis, and our work further suggested that pharmacological activation of AMPK in adipose tissue reduced hepatic glucose production via suppression of inflammation.

**Fig. 7.**
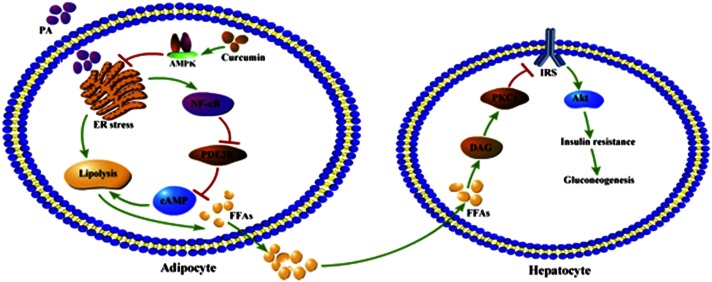
Proposed working pathways for curcumin action in inhibition of adipose lipolysis and prevention of hepatic insulin resistance. Curcumin suppressed lipolysis by attenuating ER stress via AMPK activation, leading to a suppression of FFA release from adipose tissue. Hepatic insulin resistance was prevented through the PKCε/Akt pathway as a result of lowered lipid uptake in the liver.

In summary, this study demonstrated that increased adipose lipolysis is an initial cause for the development of insulin resistance. Curcumin inhibited adipose lipolysis by blocking cAMP/PKA signaling via regulation of AMPK, and thus prevented hepatic insulin resistance by reducing DAG deposit and PKCε translocation in the liver. The proposed working pathways for curcumin action are shown in **Fig. 7**. These findings are beneficial for us to have a better understanding about curcumin action in the regulation of lipid and glucose homeostasis.

## Supplementary Material

Supplemental Data
