# Supplementary material for: Curcumin inhibits lipolysis via suppression of ER stress in adipose tissue and prevents hepatic insulin resistance
Source: J Lipid Res. 2016 Jul;57(7):1243–55. doi: 10.1194/jlr.M067397 (PMC4918853; doi:10.1194/jlr.M067397)
Supplement: Supplemental Data [file supp_57_7_1243__index.html]

Curcumin inhibits lipolysis via suppression of endoplasmic reticulum stress in adipose tissue and prevents hepatic insulin resistance — Curcumin inhibits lipolysis via suppression of ER stress in adipose tissue and prevents hepatic insulin resistance — Supplemental Data 

# Curcumin inhibits lipolysis via suppression of ER stress in adipose tissue and prevents hepatic insulin resistance

## Supplemental Data

- Supplementary Figure 1 (.pdf, 177 KB) - Mice were fed with HFD for 10 days with oral administration of curcumin (50 mg kg -1 ) or TUDCA (50 mg kg -1 ). Serum : FFAs (A), TC (B) and triglyceride (C) were determined by kit. Data were expressed as the mean ? SD (n=6).(D): Food intake; (E): Body weight gain in HFD-fed mice.
