# Supplementary material for: Curcumin inhibits lipolysis via suppression of ER stress in adipose tissue and prevents hepatic insulin resistance
Source: J Lipid Res. 2016 Jul;57(7):1243–55. doi: 10.1194/jlr.M067397 (PMC4918853; doi:10.1194/jlr.M067397)
Supplement: Supplemental Data [file 10.1194_M067397_jlr.M067397-1.pdf]

Supplementary Figure:

Figure 1

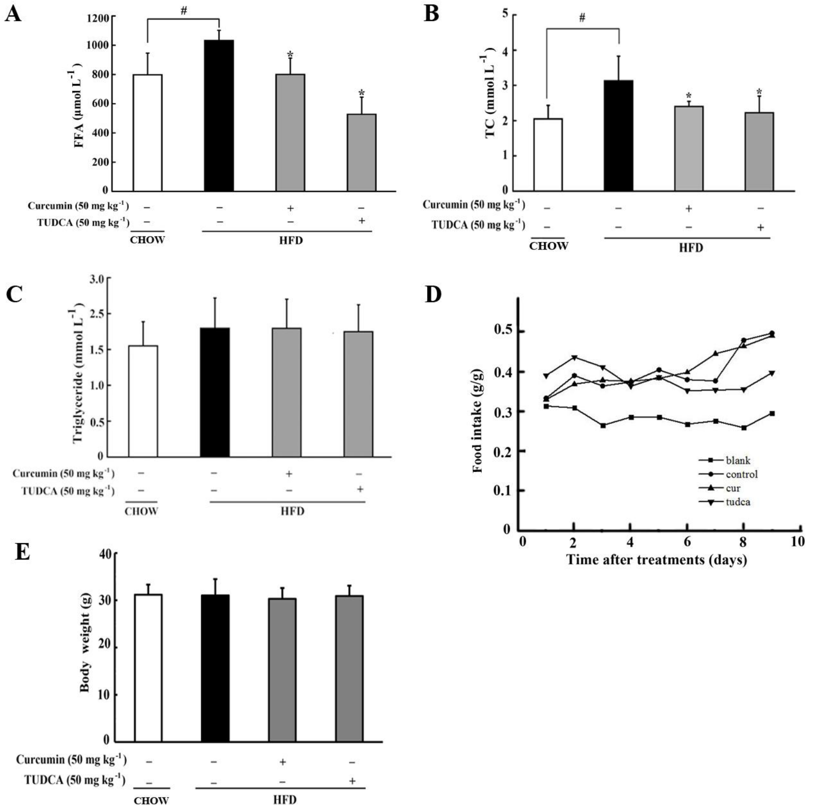

Supplementary Fig. 1 Mice were fed with HFD for 10 days with oral administration of curcumin (50 mg kg^{-1}) or TUDCA (50 mg kg^{-1}). Serum : FFAs (A), TC (B) and triglyceride (C) were determined by kit. Data were expressed as the mean ± SD (n=6). (D): Food intake; (E): Body weight gain in HFD-fed mice. The results were expressed as the mean ± SD (n=10). **p* < 0.05 *vs* model; #*p* < 0.05 *vs* control.
